# Mr. Dewar, on Cold Ablution

**Published:** 1804-01-01

**Authors:** Henry Dewar

**Affiliations:** Edinburgh


### *To the Editors of the Medical and Phyſical Journal.*

Gentlemen,

I Shall be happy if you find the following Observations, taken from the notes I kept in the Mediterranean, worthy of a place in your Journal. They relate to a subject which has of late excited some attention in the medical world, though much less than it merits; I mean, the efficacy of the cold affusion in ardent fever.

The Second, or Queen's Regiment of Foot, forming part of Sir Ralph Abercrombie's army, arrived from England at Minorca, on the 21st of July, 1800. Being sent to attend a detachment of it on board the Thisbe frigate, and afterwards doing duty with the whole regiment on shore, I had occasion to observe the facts I am now to describe.

The men enjoyed good health while on board, with the exception of two or three cases of intermittent fever, which had broke out on the passage, and a cholera morbus, which appeared in the harbour in an alarming form, though with no fatal consequences. But on the 11th of August, two days after they were landed at Mahon, an ardent fever appeared among them, which in a little time made considerable progress.

For a whole week sixteen men, on an average, were taken ill each day. Their complaints, for the most part, came on suddenly, and very often when they were on parade. After slight languor and debility, the patient was all at once seized with violent head-ach, giddiness, pains, and extreme debility in the lower extremities, rendering him totally unable either to stand or walk. When he was brought to the hospital, we found him labouring under all the symptoms of the most violent pyrexia, increased heat, quick pulse, and urgent thirst. Two or three of them had very frequently alternations of heat and cold; but, in all the rest, the preternatural heat of the skin was constant, and the patient's feelings uniformly hot and oppressive. The symptom of which they all most violently complained, was the excruciating head-ach.

I shall not trouble you with the observations I made respecting the causes of this fever, or the different remedies I employed, but confine myself to an account of the cold affusion. My opinion of its efficacy was previously fixed, from the perusal of Dr. Currie's valuable Reports, and from some striking cases which I had seen in the Edinburgh Infirmary, under the care of Dr. Gregory. I had the happiness to find that Mr. Wells, then Surgeon to the Regiment, entertained a favourable opinion of this practice, and that we had an opportunity of accommodating a great proportion of the patients in a regimental hospital under our own care. I presaged the happiest consequences from it among the men; and seized with avidity the opportunity offered, not of confirming my own belief in its advantages, but of observing such facts as might further elucidate the subject, and afford additional evidence, to convince my medical friends of the high utility of this practice.

In the mode of application, I observed the rules laid down by Dr. Currie, together with such precautions as appeared a priori to be dictated by reason. The patients to whom it was applied, were those whose skin was uniformly hotter than natural, and parched. I never used it where there was much perspiration; but, on the authority of Dr. Currie, I considered a slight moisture of the skin as forming no objection to its use. While the patient laboured in this state under tormenting head-ach, and every symptom of violent fever, I took him out of bed, stripped him quite naked, * and desiring him to hold back his head, and shut his eyes and mouth, poured a quantity of pump water first over the head, then over the breast and back, then washed the arms and palms of the hands, the thighs, legs, and soles of the feet; when the extremities, formerly hot, now

* When I had occasion to apply it to females, their delicacy was saved by allowing them to retain their shift, which was changed immediately after the operation.

now became cooler. The heat generally returned about the region of the heart; while, in the head, it continued during the first affusion with little abatement. I therefore again washed the head and breast, and so on alternately, till the whole surface became much cooler than before. After this the patient was laid in bed. If in the course of eight or ten minutes, the heat returned with equal or nearly equal intensity, he was taken out, and the operation repeated. The head-ach being the most obstinate symptom, and the last to yield to the cold affusion, the head was ordered to be shaved, and kept constantly cool, with a fold of linen laid lightly on, and dipped in water or brandy. Next day, whatever comparative relief the patient might experience, if any considerable febrile heat remained, the operation was repeated. In this application, I conceive it to be of great importance to begin with the head. The head-ach is attended with an external heat much greater in the head than over the rest of the body, indicating a peculiar force of increased action in that part of the system. To begin therefore with cooling the head, tends to restore uniformity of action through the system. The same circumstance renders it necessary to keep the head cool during the whole course of the disease.

The effect of the cold affusion thus applied was an immediate relief from the head-ach, from the heat of the skin, and all the symptoms of pyrexia. In every case the rapidity of the pulse was diminished, and the patient always felt immediate comfort. In many cases, after ten minutes a gentle perspiration broke out over the body, which still further promoted the cure. Next day, if the febrile symptoms returned, they were always milder, and a second application of the remedy greatly diminished them. The head-ach continued for some time after the other symptoms went off, but by the perpetual use of cooling applications it gradually declined, (seldom requiring the application of a blister) and left the patient with no vestige of fever, except a degree of debility. In most cases a yellow suffusion appeared over the surface in the latter stages of the disease, but it gradually went off and seemed to require no peculiarity of treatment. As medical men, in adopting a new remedy, are often too zealous for its indiscriminate use, and those authors who treat on the subject might be suspected of overlooking some of the occasional bad consequences of their practice, I made it my business to attend to the varieties of the phenomena, and to observe whether or not, in any case, this application

 was was ineffectual or seemed hazardous; but I could find no instance of this kind. Those patients who had previously a slight moisture on the skin were benefited as well as those whose skin was parched. Some shuddered and started when the water was applied, but this unpleasant sensation was very momentary, though I confess, in observing this, I would use the precaution of applying the water rather more gradually, lest an excessive shock should overpower the system in this state of febrile sensibility. The only patient in the regiment to whom the fever proved fatal, was an officer, whose obstinate disposition resisted the application of every powerful remedy, and in whom indeed the disease assumed a different type from his being subject to a constitutional gloominess of mind, increased at that time by misfortune. I attribute the general efficacy of the cold affusion on this occasion in a great measure to its early application.

A similar fever was very prevalent among the natives of the island, but still more so among the British troops, where it broke out in each regiment at a different time. From any information I could collect, the cold affusion seemed to be unknown to the physicians of the island, nor could I even find any instance in which it was employed in our military hospitals. Some of my friends advised me against its use from the unpleasant speculations to which its novelty might give rise. One gentleman, whom I in vain endeavoured to prevail on to employ it, told me afterwards that he had, notwithstanding, kept the heads of his patients cool in the same manner as I had done, and found it invariably serviceable for alleviating the head-ach. In many patients in the island the fever was attended with high delirium on the second or third day. None on whom the cold affusion was used had any delirium worthy of notice. Some of them complained of giddiness in the erect posture, and their minds were observed to waver a little during the night. From this I concluded that a strong tendency to delirium had existed, but was checked by the same remedy which removed the other symptoms. In many the fever was evidently cut short at once, and in all of them I had reason to think that its course was rendered much milder. The emaciation which appeared among the convalescents was not to be compared to that which generally takes place in fevers so violent. The disease, under this treatment, proved much less formidable than in the ships in the harbour, and in other regiments on on shore, where its fatality in some instances spread no small consternation.

Knowing the present spirit of enterprise which prevails in the medical world, I expected on my return to this country in 1802, that the cold affusion must be universally employed, and was rather mortified to find, that though no facts were brought forward to its discredit, many medical men seemed very unwilling to employ it. It is rather singular that while new articles, formerly reputed poisonous, are daily introduced into the Materia Medica, and experiments are made with them not only without scruple, but with zeal, the affusion of cold water in fever, a practice frequent among the ancients, and employed with advantage by some rude nations of modern times, should be considered as too extraordinary in its nature to receive a trial.

Some medical gentlemen complain of the prejudices of mankind as insurmountable obstacles to this practice. But do they not promote such prejudices by their mode of proceeding? They first *declare ingenuously* to the connections of the patient, that they wish to employ a remedy entirely new, a remedy which has the appearance of harshness, but on the beneficial effects of which they have considerable reliance. A preamble of this kind creates prejudices where they did not exist. The affectionate relatives are seized with the same horror as if a surgical operation on the liver or the spleen were proposed. No assurances of its safety will now prevail on them to comply with a practice, the mention of which is forced with qualms from the medical attendant, and that only in a case of extreme necessity. If it were attended with hazard, this procedure would be proper; but its apparent harshness, which arises solely from its novelty, is a very trivial objection to a practice, of the utility of which we are ourselves fully satisfied. It ought not to be mentioned, unless it occurs to the persons concerned. I see nothing to hinder any practitioner from ordering a vessel of water to the bed-side of his patient, and proceeding with the affusion without any previous ceremony. Allowing prejudices to exist, their reign will be very short. The comfort immediately experienced by the patient will quickly dissipate their uneasiness.

In the medical practice of the army, the humour of the patient is seldom consulted. But if prejudices so strong had existed, I think I should have observed them; and this I never have done. When requested on a few occasions to visit patients in private life, I have found, on mentioning ing the cold affusion, that instead of dreading it for its harshness, they were inclined to despise it for its simplicity. I recollect an aged woman under a fever in Minorca, whose daughter I prevailed on to employ it, only after prescribing as a placebo that the water should be mixed with some other ingredients. The vulgar are accustomed to place their chief reliance on drugs, of the virtues of which they are ignorant, and to overlook the effects of regimen and of temperature. Novelties in these articles do not make a forcible impression on them. But when such novelties are proposed to the physician, whose views are more extended, they appear to him in all their real importance, as changes which are not to be adopted but on the most solid foundation; and when he is at last convinced of their utility, he feels that his sentiments have undergone a serious revolution. But he should recollect how different the mode of thinking is among men at large; and that by treating other people as if their minds were affected in the same manner as his own, he may give origin to scruples which will be much less easily removed than those of an enlightened man.

It gives me very great pleasure to find from one of your late Numbers, that the cold affusion begins to be used with success in the yellow fever of North America. Where this formidable disease is attended with increased heat, all the advantages of this practice may be rationally expected. In other forms of the disease, we may probably be allowed to entertain great hopes from the employment of water of different temperatures adapted to the state of the skin, or from a judicious alternation of the hot with the cold bath. These subjects begin to be cultivated with zeal and with considerable prospect of success by professional men. In forming our practical conclusions, we must however exercise that patience and caution which subjects at once so important and so delicate of investigation demand.

I am, &c.

HENRY DEWAR.

*Edinburgh*, Nov. 14, 1803.

